# Determinants of multidrug-resistant tuberculosis in Henan province in China: a case control study

**DOI:** 10.1186/s12889-016-2711-z

**Published:** 2016-01-16

**Authors:** Chunxiao Zhang, Yongliang Wang, Guangcan Shi, Wei Han, Huayang Zhao, Huiqiang Zhang, Xiue Xi

**Affiliations:** Department of Tuberculosis, the First Hospital Affiliated to the Xinxiang Medical College, No. 88 Jiankang Road, Weihui, 453100 Henan China

**Keywords:** Tuberculosis, MDR-TB, Risk factors, Case-control

## Abstract

**Background:**

Multi-drug resistance (MDR) has been a cause of concern for tuberculosis (TB) control in both developed and developing countries. This study described the characteristics and risk factors associated with MDR-TB among 287 cases and 291 controls in Henan province, China.

**Methods:**

A hospital-based case-control study was conducted between June 2012 and December 2013. The study subjects were selected using multistage probability sampling. Multivariate conditional logistic regression models were used to determine the risk factors associated with MDR-TB.

**Results:**

The following risk factors for MDR-TB were identified: previous TB treatment (AOR = 4.51, 95 % CI: 3.55–5.56), male sex (AOR = 1.09, 95 % CI: 0.24–1.88), high school or lower education degree (AOR = 1.87, 95 % CI: 1.27–2.69), unemployment (AOR = 1.30, 95 % CI: 0.78–2.52), long distance of residence from the health facility (AOR = 6.66,95 % CI: 5.92–7.72), smoking (AOR = 2.07, 95 % CI: 1.66–3.19), poor knowledge regarding MDR-TB (AOR = 2.06, 95 % CI: 1.66–2.92), traveling by foot to reach the health facility (AOR = 1.85, 95 % CI: 1.12–3.09), estimated amount of time to reach the health facility was greater than 3 h (AOR = 1.42, 95 % CI: 0.51–2.35), social stigma (AOR = 1.17, 95 % CI: 0.27–2.03), having an opportunistic infection (AOR = 1.45, 95 % CI: 0.58–2.4), more than 3 TB foci in the lungs (AOR = 1.98, 95 % CI: 1.49–3.25), total time of first treatment was more than 8 months (AOR = 1.39, 95 % CI: 0.65–2.54), adverse effects of anti-TB medication (AOR = 2.39, 95 % CI: 1.40–3.26), and more than 3 prior episodes of anti-TB treatment (AOR = 1.83, 95 % CI: 1.26–2.80).

**Conclusion:**

The identified risk factors should be given priority in TB control programs. Additionally, there is a compelling need for better management and control of MDR-TB, particularly through increasing laboratory capacity, regular screening, enhancing drug sensitivity testing, novel MDR-TB drug regimens, and adherence to medication.

## Background

Tuberculosis (TB), which is an infectious airborne disease, is a major health problem worldwide. Globally, TB is the second most lethal disease, next only to AIDS [1, 2]. Additionally, a growing concern is the development of multi drug resistant (MDR) forms of TB, which have developed due to partial or incomplete treatments. MDR-TB is defined as disease with mycobacterial strains that are resistant to two of the most effective and important anti-TB drugs: isoniazid and rifampin. These two drugs are considered first-line drugs and are recommended for the treatment of all individuals with drug-susceptible TB disease.

In China, 6 % of *Mycobacterium tuberculosis* isolates are resistant to isoniazid, and 1 % of isolates are multidrug resistant [3]; thus, MDR-TB has become a major public health problem and an obstacle to global TB control [4]. Globally, more than 10.5 million incident cases of TB were estimated to occur in 2010, most of which occurred in Asia and Africa [5]. The mortality rates due to MDR-TB have appeared to increase in leaps and bounds, and this ominous phenomenon is generally observed in developing and under-developed parts of the world [6, 7]. Overall, the 27 high burdened countries account for 85 % of all MDR-TB cases. In light of the rising numbers of TB cases, the problem of anti-tubercular drug resistance remains a significant concern [8]. In China, MDR-TB cases are more difficult and costly to treat and require appropriate treatment and control mechanisms [9]. Although TB is one hundred percent curable, MDR-TB is more difficult to treat than drug susceptible TB because it requires the use of less effective second line anti tubercular drugs, which are often associated with major side effects [10].

Currently, no epidemiological or case control studies are available in Henan province for MDR-TB. Therefore, to describe the characteristics and assess the factors that determine the occurrence of MDR-TB in Henan province, we conducted a case control study on MDR-TB between June 2012 and December 2013.

## Methods

### Ethical consideration

Ethical clearance (2012HN12254, June 2012-December 2013) was obtained from the medical ethics committee of the medical association of Henan province and from each selected health facility. During the study, ethical clearance was also obtained from the health facilities on behalf of the study participants to collect secondary data and to trace the study participants who were not available at the clinic during the study period. Voluntary written informed consent was obtained from all of the participants included in the study.

### Study design and study area

This case-control study was conducted from June 2012 to December 2013. The study was conducted in Henan province, China. Henan province has 18 cities and 89 towns, 342 administrative villages, and an estimated population of one hundred million; 69.4 % of the inhabitants live in rural areas. This case-control study among MDR-TB patients was conducted retrospectively.

### Study population

For the present study, the MDR-TB cases were selected from the following five hospitals: the first affiliated hospital of Xinxiang medical college, the Xinxiang city people’s hospital, the TB hospital in Henan province, the Xinxiang city huaxin hospital, and the Xinxiang city second people’s hospital. The controls were selected from the following five community health service centers in Xinxiang city: the Jian She road community health service center, the Yan Dian street community health service center, the De Bei street community health service center, the Xin Zhuang street community health service center, and the De Nan street community health service center.

The cases were selected using multistage probability sampling methods, and the controls were selected by random sampling from the same five hospitals. The inclusion criteria for the cases were as follows: (1) *M. tuberculosis* was detected in the patient’s bacterium identification test; (2) the strains were resistant to at least isoniazid and rifampin by drug susceptibility testing, which was routinely performed on isolates of *M. tuberculosis* using the proportion method (minimum inhibitory concentrations: isoniazid 0.2 mg/ml, rifampin 40 mg/ml, streptomycin 4 mg/ml, ethambutol 2 mg/ml, kanamycin 30 mg/ml, and ofloxacin 2 mg/ml); (3) a history of treatment with anti-TB drugs for more than 1 month, i.e., treatment classification in the medical records was ‘previously treated’, including relapses, treatment after failure, treatment after default, and chronic patients; (4) the patient was registered between June 2012 and December 2013; (5) the patient was willing to participate in the study; and (6) complete medical records were available; (7) the cases of primary MDR (untreated patients) were exluded.

The inclusion criteria for the controls were the same as those for the cases, except that the strains were sensitive to both isoniazid and rifampin by drug susceptibility testing. The exclusion criterion for the cases and controls was as follows: the patient could not provide correct information, such as TB patients with mental illness or alcohol abuse patients.

### Drug susceptibility test

Sputum samples were processed using *N*-acetyl-L-cysteinesodium hydroxide and centrifugally concentrated for acid fast bacilli smear examination and *M. tuberculosis* culture. Drug susceptibility testing was performed as previously [11]. Briefly, the concentrated pellet was suspended in buffer (pH 6.8) and was cultured in liquid medium (BACTEC MGIT 960, Becton Dickenson Diagnostics). A Middlebrook 7H10 agar (Difco), 1 % proportion test of susceptibility to isoniazid 0.2 μg/ml, rifampin 1 μg/ml, streptomycin 2 μg/ml, ethambutol 5 μg/ml, kanamycin 5 μg/ml, and ofloxacin 4 μg/ml was performed on *M. tuberculosis* confirmed cultures. Patients resistant to isoniazid and rifampin (with or without resistance to additional anti-TB drugs) were classified as MDR-TB infected.

### Data collection

A structured questionnaire was used to collect information from the study participants. Secondary data were collected from TB and MDR-TB registers. Patient charts and the data collection format were used to determine and record the patients’ initial TB episode. Day-long training was provided to the nurses and health officers involved in the data collection process. The main variables included in study instrument were sex, age, nationality, religion, occupation, education degree, marital status, monthly income of the family (RMB), patient residence, family size, estimated distance of residence from the health facility, smoking, alcoholism, knowledge regarding MDR-TB, means of transportation to reach the health facility, estimated time to reach the health facility (hrs), social stigma, previous TB treatment, previous known contact with an MDR-TB patient, opportunistic infection, defaulting from treatment, number of TB foci in the lung, total time of first treatment, adverse effects of anti-TB medication, and prior episodes of anti-TB treatment.

### Data management and analysis

The data were entered using Epidata version 3.1, and the data analysis was performed using SPSS 16.0 software (SPSS Inc., Chicago, IL, USA). The data completeness and consistency were checked by running frequencies of each variable. Multiple logistic regression models were used to control the confounding effects of different variables while assessing the effect of each variable on the likelihood of MDR-TB occurrence. The odds ratios (ORs) and 95 % confidence intervals (95 % CIs) were used to quantify the strength of the association between potential risk factors and MDR-TB. A *p*-value of 0.05 was used as the cut-off point for statistical significance.

## Results

### Socio-demographic characteristics of the study participants

The analyses of socio-demographic characteristics of participants (including sex, age, nationality, religion, occupation, education, marital status, family income, patient residence and family size) are shown in Table 1.

**Table 1:** Socio-demographic distribution of cases and controls, Henan province, 2013

Variables		Case (287)		Control (291)		Total (578)	
		*n*	%	*n*	%	*n*	%
Sex							
	Female	58	20.21	89	30.58	147	25.43
	Male	229	79.79	202	69.42	431	74.57
Age (years)							
	≤29	39	13.59	29	9.96	68	11.76
	30–59	64	22.3	68	23.37	132	22.84
	≥60	184	64.11	194	66.67	378	65.4
Nationality							
	Han Nationality	251	87.34	241	82.75	492	85.12
	Other Nationality	36	12.66	50	17.25	86	14.88
Religion							
	Christian	20	7.12	15	5.32	35	6.05
	Muslim	7	2.42	9	2.96	16	2.77
	No religion	260	90.46	267	91.72	527	91.18
Occupation							
	Unemployed	182	63.41	151	51.89	333	57.61
	Employed	105	36.59	140	48.11	245	42.39
Education degree							
	High school or lower	211	73.52	157	53.95	368	63.67
	University or higher	76	26.48	134	46.05	210	36.33
Marital status							
	Married	185	64.57	179	61.39	364	62.98
	Single	76	26.48	72	24.83	148	25.61
	Divorced	20	6.84	31	10.52	51	8.82
	Widowed	6	2.11	9	3.26	15	2.59
Monthly income of the Family (RMB)							
	≤3000	231	80.44	228	78.35	459	79.41
	3000–5000	45	15.57	40	13.89	85	14.71
	≥5000	11	3.99	23	7.76	34	5.88
Patient residence							
	Rural	197	68.49	175	60.18	372	64.36
	Urban	90	31.51	116	39.82	206	35.64
Family size							
	≤3	142	49.32	156	53.74	298	51.56
	≥4	145	50.68	135	46.26	280	48.44

### Factors associated with MDR-TB

There was a very large difference in previous TB treatment history between the cases and controls: 214 (74.36) cases had previously been treated for TB, whereas only 127 (43.82) controls had a previous TB treatment history. As a result, previous TB treatment (AOR = 4.51, 95 % CI: 3.55–5.56) was found to be a strong risk factor for MDR-TB. In addition, this study revealed that the male sex (AOR = 1.09, 95 % CI: 0.24–1.88), a high school or lower education degree (AOR = 1.87, 95 % CI: 1.27–2.69), unemployment (AOR = 1.30, 95 % CI: 0.78–2.52), long distance of residence from the health facility (AOR = 6.66, 95 % CI: 5.92–7.72), having ever smoked (AOR = 2.07, 95 % CI: 1.66–3.19), poor knowledge regarding MDR TB (AOR = 2.06, 95 % CI: 1.66–2.92), traveling by foot to reach the health facility (AOR = 1.85, 95 % CI: 1.12–3.09), estimated amount of time to reach the health facility was greater than 3 h (AOR = 1.42, 95 % CI: 0.51–2.35), social stigma (AOR = 1.17, 95 % CI: 0.27–2.03), having an opportunistic infection (AOR = 1.45, 95 % CI: 0.58–2.4), more than 3 TB foci in the lung (AOR = 1.98, 95 % CI: 1.49–3.25), first treatment lasted more than 8 months (AOR = 1.39, 95 % CI: 0.65–2.54), adverse effects of anti-TB medication (AOR = 2.39, 95 % CI: 1.40–3.26), and more than 3 prior episodes of anti-TB treatment (AOR =1.83, 95 % CI: 1.26–2.80) as the most important factors contributing to the development of MDR-TB in Henan province in China. However, this study did not indicate that age, alcoholism, ventilation in room, having previous known contact with an MDR-TB patient, or defaulting from treatment were significantly associated with MDR-TB development (see Table 2).

**Table 2:** Determinants of multidrug-resistant tuberculosis from the logistic regression model, Henan province, China, 2013

Variable	Case (*n* = 287)	Control (*n* = 291)	COR (95 % CI)	AOR (95 % CI)^a^	*P* Value
Sex					
Male	229 (79.79)	202 (69.42)	0.57 (1.43–2.76)	1.09 (0.24–1.88)	0.006
Female	58 (20.21)	89 (30.58)	1		
Age (years)					
≤29	39 (13.35)	29 (10.14)	1.43 (0.53–2.81)	2.08 (1.11–2.94)	0.401
30–59	64 (22.43)	68 (22.85)	0.99 (1.18–2.71)	1.48 (0.62–2.26)	0.642
≥60	184 (64.22)	194 (67.01)	1		
Education degree					
High school or lower	211 (73.55)	157 (54.16)	2.37 (1.49–4.23)	1.87 (1.27–2.69)	0.000
University or higher	76 (26.45)	134 (45.84)	1		
Occupation					
Unemployed	182 (63.18)	151 (51.76)	1.61 (0.55–4.05)	1.30 (0.78–2.52)	0.007
Employed	105 (36.81)	140 (48.24)	1		
Estimated distance of residence from the health facility					
Long	236 (82.19)	126 (43.52)	6.06 (5.04–7.85)	6.66 (5.92–7.72)	0.000
Short	51 (17.81)	165 (56.48)	1		
Smoking					
Ever	212 (73.58)	143 (49.38)	2.93 (1.98–4.36)	2.07 (1.66–3.19)	0.000
Never	75 (26.42)	148 (50.62)	1		
Alcoholism					
Yes	113 (39.16)	125 (43.25)	0.86 (0.37–2.03)	0.43 (0.37–1.42)	0.429
No	174 (60.84)	166 (56.75)	1		
Ventilation in Room					
Yes	139 (48.37)	146 (50.29)	0.93 (0.62–1.93)	1.55 (0.65–2.52)	0.738
No	148 (51.63)	145 (49.71)	1		
Knowledge on MDR-TB					
Poor	211 (73.48)	143 (49.42)	2.87 (2.46–3.88)	2.06 (1.66–2.92)	0.000
Good	76 (26.52)	148 (50.58)	1	1	
Means of transportation to reach the health facility					
On foot	175 (61.29)	143 (49.46)	1.62 (1.38–3.43)	1.85 (1.12–3.09)	0.006
By car	112 (38.71)	148 (50.54)	1	1	
Estimated time it takes to reach the health facility (hrs)					
>3	142 (49.67)	152 (52.46)	0.9 (0.44–2.54)	1.42 (0.51–2.35)	0.000
≤3	145 (50.33)	139 (47.54)	1	1	
Social Stigma					
Yes	183 (63.67)	140 (48.27)	1.9 (1.47–4.12)	1.17 (0.27–2.03)	0.000
No	104 (36.33)	151 (51.73)	1	1	
Previous TB treatment					
Yes	214 (74.36)	127 (43.82)	3.79 (3.36–5.35)	4.51 (3.55–5.56)	0.000
No	73 (25.64)	164 (56.18)	1	1	
Having previous known contact with an MDR-TB patient					
Yes	170 (59.16)	122 (42.09)	2.01 (1.77–3.65)	1.37 (0.61–2.16)	0.562
No	117 (40.84)	169 (57.91)	1	1	
Having an opportunistic infection					
Yes	185 (64.37)	147 (50.68)	1.78 (1.28–3.55)	1.45 (0.58–2.4)	0.001
No	102 (35.63)	144 (49.32)	1	1	
Defaulting from treatment					
Yes	145 (50.18)	160 (55.28)	0.84 (0.09–3.04)	0.28 (0.38–1.18)	0.322
No	142 (49.82)	131 (44.72)	1	1	
Number of TB foci in the lung					
>3	172 (59.83)	143 (49.27)	1.55 (1.13–3.72)	1.98 (1.49–3.25)	0.012
≤3	115 (40.17)	148 (50.73)	1	1	
Total length of first treatment					
≥8 months	157 (54.38)	120 (41.28)	1.72 (1.02–3.49)	1.39 (0.65–2.54)	0.001
<8 months	130 (45.62)	171 (58.72)	1	1	
Adverse effects of anti-TB medication					
Yes	177 (61.47)	148 (50.87)	1.55 (1.26–3.14)	2.39 (1.40–3.26)	0.011
No	110 (38.53)	143 (49.13)	1	1	
Prior episodes of anti-TB treatment					
≥3 times	176 (61.26)	152 (52.38)	1.45 (0.7–3.16)	1.83 (1.26–2.80)	0.034
<3 times	111 (38.74)	139 (47.62)	1	1	

*Note*. ^a^Adjusted for age, sex

## Discussion

Anti-TB drug resistance is a major public health problem that threatens progress in TB care and control worldwide. Essentially, drug resistance arises in areas with weak TB control programs [12]. TB bacteria can become resistant to the drugs used for treatment. When this occurs, treatment is often still possible, but it is complex, long, challenging, and expensive [10]. Furthermore, a patient who develops active disease with a drug-resistant TB strain can transmit this form of TB to other individuals. Drug-resistant TB can occur when the drugs used to treat TB are not used as prescribed. This may happen when: a patient misses doses or does not complete the full course of treatment; a health care provider prescribes the wrong treatment, wrong dose, or wrong length of time for taking the drugs; effective drugs are not available; and the drugs are of poor quality [7, 13]. Some theoretical studies have suggested that drug resistance in *M. tuberculosis* occurs due to genetic factors, factors related to previous anti-TB treatment, human immunodeficiency virus (HIV) co-infection and environment factors [1, 14]. Although there is some evidence to postulate host genetic predisposition as the basis for the development of MDR-TB, the accumulation of changes in the genomic content that occur through gene acquisition and loss is the major underlying event in the emergence of fit and successful strain variants in the *M. tuberculosis* complex. Spontaneous chromosomally borne mutations occurring in *M. tuberculosis* at a predictable rate are thought to confer resistance to anti-TB drugs [15].

Previous studies found that being male was a risk factor for MDR-TB development. Similarly, studies showed that the male sex was a risk factor for the development of MDR-TB [15, 16]. Another study showed that males have an increased risk for MDR-TB, and being male was a risk factor for defaulting from anti-TB medication. Moreover, this study showed that among MDR-TB cases who were defaulters in their first-line TB treatment, 62.5 % were males [15]. Likewise, our study showed that males had an increased risk for the development of MDR-TB. The association between being male and having MDR-TB could be because males have a higher tendency to not adhere to anti-TB treatment than females, thus increasing their risk of developing MDR-TB [17].

Marital status, as one kind of sociodemographic characteristic, is often related to diseases, including TB. In our study, being single was a risk factor for MDR-TB, which was in agreement with many other studies that showed that single persons were more likely to develop TB [18, 19]. Although there isn’t an identifiable biological relationship between marital status and TB, single persons are more likely to lack social support or be involved in high-risk behaviors, such as alcohol consumption or smoking, which potentially leaves them exposed to higher risks of infection of TB or MDR-TB than those with other marital statuses.

Like marital status, income levels are also interrelated with disease and health. Previous studies found that a low socioeconomic status or poverty were associated with MDR-TB [20, 21]. In our study, low income was also a risk factor associated with MDR-TB. Consequently, we infer that it is easy for TB patients in low-income communities to acquire MDR-TB because there is a lack of good medical services. With the increase of acquired MDR-TB patients in low-income communities, the general population has higher chances of contracting MDR-TB. This finding also suggests that transmission of MDR-TB is not extensive in the general population and still confined to low-income communities.

In this study, we first identify social stigma as risk factors for MDR-TB in China, with no identifiable risk factor reported prior to this. It is known that bad habits potentially increase the risks for MDR-TB. If bad habits develop into crime leading to social stigma, it might become a risk factor associated with TB. Therefore, our study provided important evidence that social stigma was a risk factor for MDR-TB.

Another study assessing the risk factors associated with MDR-TB revealed that previous treatment was the strongest determinant of MDR-TB and that the pooled risk of MDR-TB was more than 5 times higher in previously treated cases than in never-treated cases [22]. As found in many other studies, a history of anti-TB treatment has been consistently associated with the risk of MDR-TB. Previously treated patients often constitute a very heterogeneous group including those who experience relapse after receiving successful treatment, those who return after default, and those who start receiving a re-treatment regimen after having experienced previous treatment failure [9]. Our study is consistent with former reports that revealed the importance of previous treatment history in the risk of drug resistance.

The principal patient related factor that predicts the occurrence of MDR-TB is drug non adherence; thus, the category II regimen was blamed for increasing the chances of developing MDR-TB. MDR-TB develops due to errors in TB management such as the use of a single drug to treat TB, the addition of a single drug to a failing regimen, the failure to identify preexisting resistance, the initiation of an inadequate regimen using first line anti tubercular drugs and variations in the bioavailability of anti-TB drugs to predispose the patient to the development of MDR-TB [23]. A shortage of drugs has been one of the most common reasons for the inadequacy of the initial anti-TB regimen, particularly in resource poor settings [24]. Other major issues significantly contributing to the higher complexity of the treatment of MDR-TB is the increased cost of treatment.

Certainly, inadequate treatment adherence also increases the risk of developing MDR-TB. Non-adherence to prescribed treatment is often underestimated by the physician and is difficult to predict. Certain factors such as psychiatric illness, alcoholism, drug addiction, and poverty can predict non-adherence to treatment [25]. Poor compliance with treatment is also an important factor in the development of acquired drug resistance [26]. MDR-TB requires a two- to four-fold longer period of treatment compared with drug susceptible TB [15]. Most of the problems from which drug resistance originates are related to length of treatment. The longer time that is required to treat MDR-TB clearly implies an additional risk of poor treatment adherence and consequently of treatment failure [1, 13].

Other factors also play important roles in the development of MDR-TB, such as poor administrative control on the purchase and distribution of the drugs with no proper mechanisms for quality control and bioavailability tests [27]. TB control programs implemented in the past have also partially contributed to the development of drug resistance due to poor follow up and infrastructure.

Therefore, the following recommendations should be considered: health professionals ought to enhance their efforts in controlling MDR-TB; public awareness regarding MDR-TB should be increased; the effectiveness of category II regimens in treating TB patients requires further study; better management and control of TB, particularly drug resistant TB, should be conducted by experienced and qualified doctors; access to standard microbiology laboratories is necessary; understanding the co-morbidity of HIV and TB is necessary; new anti-TB drug regimens and better diagnostic tests should be created; the laboratory capacity for drug sensitivity tests should be increased as much as possible; international standards should be created for second line drug-susceptibility testing; new antitubercular molecules and vaccines should be invented, and we must determine the real magnitude of MDR-TB. These recommendations are some of the important issues that must be addressed for effective prevention, control and management of MDR-TB. The strengths of the current study are that the cases and controls had a better likelihood of coming from similar backgrounds and had most likely received the same services. All of the cases that fulfilled the eligibility criteria who were available during the study period and willing to respond were included in the study. This was helpful in decreasing sampling error.

However, the current study is not without limitations. Recall bias could be considered a potential challenge because some of the information was based on the recall of the study participants. Furthermore, there is also a potential bias in estimating drug resistance in patients. Although we reviewed the medical records and screened the case notification system to check for previous episodes of TB, we could not exclude the possibility of misclassification. Moreover, because of the retrospective nature of our study, it was not possible to demonstrate a causal relationship between the variables. Additionally, missing data may have affected our results. Nevertheless, our findings are similar to previous reports. Thus, we believe that the effect of missing values was not important in our analysis, and we have adjusted our results according to some confounding factors (sex and age). Although our primary interest was to identify risk factors that increased the risk of developing MDR-TB associated with the occurrence of anti-TB adverse drug reactions, these findings need to be corroborated with multi-center case-control studies to bring out nationally relevant risk factors for MDR-TB.

## Conclusion

In conclusion, previous TB treatment, male sex, low education, unemployment, smoking, poor knowledge of MDR TB, social stigma, adverse effects of anti-TB medication, prior episodes of anti-TB treatment, being far from the health facility, traveling to the health facility on foot, and more TB foci were shown to increase the risk for MDR-TB. The identified risk factors should be given priority in effective TB control programs under the health bureau of Henan province to control MDR-TB. Evaluating the current scenario, there is a compelling and urgent need for increasing public awareness, effective new anti-TB drug regimens, standard microbiology laboratories, better diagnostic tests, enhancing drug sensitivity tests, inventing new antitubercular molecules and vaccines, regular screening, and better management and control of MDR-TB; these actions will be stepping stones towards controlling this disease, which is becoming deadlier by the day.
